# PReS-FINAL-2080: Overlap syndrome between primary sclerosing cholangitis and autoimmune hepatitis associated with ulcerative colitis - a case of unusual presentation in a patient with systemic juvenile idiopathic arthritis

**DOI:** 10.1186/1546-0096-11-S2-P92

**Published:** 2013-12-05

**Authors:** M Cabral, S Batalha, J Nunes, R Jotta, M Cravo, P Oom

**Affiliations:** 1 Pediatrics, Hospital Beatriz Ângelo, Loures, Portugal; 2 Gastroenterology, Hospital Beatriz Ângelo, Loures, Portugal

## Introduction

Systemic juvenile idiopathic arthritis (systemic-JIA) is characterized by the presence of arthritis associated with systemic manifestations and an important inflammatory response, being always a clinical diagnosis of exclusion. The association of systemic-JIA with autoimmune diseases is uncommon. The authors found only one description of systemic-JIA associated with autoimmune hepatitis in a child treated with etanercept.

## Objectives

Not indicated (case report).

## Methods

Not indicated (case report).

## Results

Case report: 16-year-old male adolescent, hospitalized for investigation of high fever with two weeks of evolution, accompanied by chest pain, right elbow and knee arthritis, jaundice and generalized pruritus. Laboratory findings: haemoglobin-9,3mg/dL, 582.000 platelets/uL, ESR-107mm/1^st ^hr, C-reactive protein-11,75mg/dL, AST-246U/L, ALT-436U/L, alkaline phosphatase-708U/L, G-GT-702U/L, total/direct bilirrubin-2,61/2,57mg/dL, G and A immunoglobulin levels elevation. Screening for infectious, autoimmune and lymphoproliferative etiologies was negative. Abdominal ultrassonography revealed an homogeneous mild hepatosplenomegaly, and thoraco-abdominal CT identified small pleural and pericardial effusion. Gathering diagnostic criteria for systemic-JIA he was treated with oral prednisolone, with further progressive and complete clinical remission, keeping, however, persistent aminotransferases levels elevation (AST-265 U/L, ALT-532 U/L) and G-GT (711 U/L) with posterior positivity for ANA (1/640), persisting negative all other autoantibodies. MR-cholangiopancreatography was performed (without changes of intrahepatic or extrahepatic bile ducts), and liver biopsy histology was compatible with primary sclerosing cholangitis (PSC) meeting, however, clinical criteria for autoimmune hepatitis (AIH). Four months after the initial diagnosis, in the context of diarrhea and hematochezia, it was performed a colonoscopy, which revealed macroscopic mucosal abnormalities suggestive of ulcerative colitis (UC), subsequently confirmed by histopathology findings. Presently, the patient is clinically stable on therapy with prednisolone, azathioprine, ursodeoxycholic acid and mesalazine.

## Conclusion

The autoimmune liver diseases are responsible for up to 5% of cases of chronic liver disease in children, and includes the AIH and PSC. When clinical and/or histological findings suggest both entities being present in the same patient, at diagnosis or during disease's evolution, is considered the diagnosis of overlap syndrome (OS). The association of UC with PSC is well established in the literature and may occur in up to 80% of patients. Although there are described associations between autoimmune liver diseases and other autoimmune diseases, this is, to our knowledge, the first case reported of systemic-JIA, in association with OS. This association raises important questions regarding the origin and complexity of autoimmune diseases in children, and demonstrates the difficulty in diagnosis and treatment imposed by these diseases.

## Disclosure of interest

None declared.

